# Comparison of In-Hospital Outcomes of Acute ST Segment Elevation Myocardial Infarction in Young Versus Older Patients

**DOI:** 10.7759/cureus.111767

**Published:** 2026-06-29

**Authors:** Yasotha Gnanakaran, Muhammad Hassaan Qayyum, Samar Raza, Hafiz Zunair Iqbal, Saba Arshad Ali, Umaima Jawad, Ammar Noor, Muhammad Ayoob Memon, FNU Qurat-Ul-Ain, Muhammad Irfan Jamil

**Affiliations:** 1 Cardiology, Blackpool Victoria Hospital, Blackpool, GBR; 2 Internal Medicine, Combined Military Hospital (CMH) Lahore Medical College and Institute of Dentistry, Lahore, PAK; 3 Diabetes and Endocrinology, Blackpool Victoria Hospital, Blackpool, GBR; 4 Plastic Surgery, Greater Lancashire Hospital, Preston, GBR; 5 Internal Medicine, Shalamar Medical & Dental College, Lahore, PAK; 6 Internal Medicine, Akhtar Saeed Medical and Dental College, Lahore, PAK; 7 Cardiology, Fatima Memorial Medical and Dental College, Lahore, PAK; 8 Cardiology, Chandka Medical College, Larkana, PAK; 9 Internal Medicine, Lahore General Hospital, Lahore, PAK; 10 Internal Medicine, Jinnah Sindh Medical University, Karachi, PAK; 11 Internal Medicine, Surgi Care Hospital, Dera Ghazi Khan, PAK; 12 Nephrology, Lahore General Hospital, Lahore, PAK; 13 Internal Medicine, Mayo Hospital, Lahore, PAK

**Keywords:** acute kidney injury, cardiogenic shock, in hospital mortality, mechanical complication, st-elevation myocardial infarction (stemi)

## Abstract

Background

Acute ST-segment elevation myocardial infarction remains a major cause of early hospital complications and death. Although it is more common in older patients, young adults are increasingly presenting with ST-segment elevation myocardial infarction (STEMI). Age may influence risk factors, clinical presentation, coronary anatomy, treatment response, and short-term outcomes, making comparison between young and older patients clinically important. This study aimed to compare in-hospital outcomes of acute ST-segment elevation myocardial infarction between young and older patients and identify independent predictors of in-hospital mortality.

Methods

This prospective analytical observational study was conducted in the Department of Cardiology at a tertiary care hospital in Lahore, over 12 months. A total of 300 patients with acute STEMI presenting within 12 hours of symptom onset were enrolled by non-probability consecutive sampling and divided into young patients aged ≤40 years and older patients aged >40 years, with 150 patients in each group. Demographic characteristics, cardiovascular risk factors, clinical presentation, laboratory profile, electrocardiogram (ECG), echocardiographic and angiographic findings, reperfusion details, procedural variables, and in-hospital outcomes were recorded. Data were analyzed using SPSS version 26.0 (IBM Corporation, Armonk, USA); Chi-square, Fisher’s exact, independent samples t-test, Mann-Whitney U test, and multivariable logistic regression were applied.

Results

Of 300 patients with STEMI, 150 were included in each age group. Young patients were more frequently male than older patients, 131 (87.3%) versus 116 (77.3%) (p=0.023), and had higher smoking frequency, 86 (57.3%) versus 53 (35.3%) (p<0.001), and a family history of cardiovascular disease, 36 (24.0%) versus 16 (10.7%) (p=0.002). Older patients had a greater comorbidity burden, including higher diabetes, hypertension, dyslipidemia, cerebrovascular events, and chronic kidney disease (all p<0.001), and more dyspnea, 73 (48.7%) versus 34 (22.7%) (p<0.001). Anterior STEMI, 85 (56.7%) versus 57 (38.0%) (p=0.001), left anterior descending artery (LAD) involvement, 81 (54.0%) versus 58 (38.7%) (p=0.008), and single-vessel disease, 107 (71.3%) versus 79 (52.7%) (p=0.001), were more frequent in young patients, whereas triple-vessel disease was higher in older patients, 26 (17.3%) versus 9 (6.0%) (p=0.002). Older patients had higher heart failure, 40 (26.7%) versus 16 (10.7%) (p<0.001), cardiogenic shock, 21 (14.0%) versus 7 (4.7%) (p=0.006), acute kidney injury (AKI), 24 (16.0%) versus 7 (4.7%) (p=0.001), mechanical complications, 19 (12.7%) versus 5 (3.3%) (p=0.003), and in-hospital death, 17 (11.3%) versus 6 (4.0%) (p=0.017). On multivariable analysis, mechanical complications [adjusted odds ratio (AOR) 13.13; p<0.001], ventricular arrhythmias (AOR 10.57; p<0.001), cardiogenic shock (AOR 6.25; p=0.010), and AKI (AOR 4.84; p=0.028) independently predicted in-hospital mortality, while the older age group was not independently associated with mortality (p=0.801).

Conclusion

Older STEMI patients had more adverse in-hospital outcomes, mainly due to acute complications and comorbidity burden. Mortality was independently related to mechanical complications, ventricular arrhythmias, cardiogenic shock, and AKI rather than age group alone.

## Introduction

Acute ST-segment elevation myocardial infarction (STEMI) is a time-sensitive cardiac emergency caused by acute coronary occlusion, leading to myocardial ischemia and necrosis when reperfusion is delayed [1]. It remains an important cause of hospital admission, short-term complications, and cardiovascular death despite wider use of primary percutaneous coronary intervention and fibrinolytic therapy. Cardiovascular disease remains a major global health problem, with more than 17 million deaths annually, and it accounts for nearly one-third of all global deaths [2,3]. In Pakistan, cardiovascular disease accounted for 25.3% of all deaths in 2019, and the national burden is influenced by smoking, unhealthy diet, physical inactivity, hypertension, environmental exposure, and unequal access to preventive care [4,5]. Although STEMI is more frequent among older adults, younger patients are increasingly recognized in hospital practice. Age is clinically relevant because it may influence baseline risk profile, symptom pattern, coronary anatomy, treatment delay, reperfusion response, and early complications. Therefore, age-based comparison of STEMI outcomes remains important for risk assessment and hospital management planning [6,7].

Previous evidence suggests that young STEMI patients usually have higher smoking exposure, male predominance, family history, and dyslipidemia, whereas older patients more often have diabetes, hypertension, renal disease, and diffuse coronary artery involvement [8,9]. Older patients have been reported to experience higher rates of heart failure, cardiogenic shock, acute kidney injury, mechanical complications, and mortality, while younger patients generally have lower in-hospital mortality but may remain at risk of recurrent myocardial infarction and repeat revascularization [10,11]. Reported findings vary across studies because of differences in age cutoffs, hospital setting, reperfusion access, symptom-to-treatment time, and baseline disease severity [2,5,10,11].

This study was needed because age-based STEMI comparisons from regional settings may not fully reflect the patient profile, treatment pathway, and hospital outcomes seen in local tertiary cardiac care. A structured comparison can help identify which risk factors and early clinical features are more common in young and older patients, and which complications are most closely related to in-hospital death. Such information may support earlier recognition of high-risk presentations, rational monitoring, renal protection, timely reperfusion, and appropriate secondary prevention. The study also allows assessment of whether older age itself remains associated with mortality after accounting for acute complications. Therefore, the present study was conducted to determine in-hospital outcomes and assess their association with demographic, clinical, laboratory, electrocardiogram (ECG), echocardiographic, angiographic, and treatment-related variables among patients presenting with acute STEMI.

## Materials and methods

After approval from the Institutional Review Board (IRB No. 127/RC/KEMU), this prospective analytical observational study was conducted in the Department of Cardiology at Mayo Hospital, Lahore, over 12 months from December 2024 to December 2025. Patients were enrolled by non-probability consecutive sampling after written informed consent. A sample size of 300 patients, 150 in each group, was calculated by using a two-proportion formula in MedCalc Statistical Software (MedCalc Software Ltd, Ostend, Belgium), taking mechanical complications as 14% in older patients and 2% in young patients [12]. Patients were divided into two groups according to age: young patients, aged ≤40 years, and older patients, aged >40 years. Patients aged 18 to 75 years with acute ST-segment elevation myocardial infarction presenting within 12 hours of symptom onset were included. Patients with previous coronary artery bypass grafting, non-ST elevation myocardial infarction (MI), cardiomyopathy, valvular heart disease, congenital heart disease, severe liver or kidney disease, systemic infection, pregnancy, lactation, drug or alcohol abuse, or any condition limiting life expectancy or interfering with cardiac intervention were excluded.

Acute STEMI was defined by acute chest pain or equivalent symptoms with ST-segment elevation in two or more contiguous ECG leads, with or without new left bundle branch block or true posterior MI, supported by cardiac biomarkers. Baseline data were recorded on a structured proforma, including age, gender, weight, height, body mass index (BMI), smoking history in pack-years, educational status, diabetes mellitus, hypertension, chronic kidney disease, dyslipidemia, family history of cardiovascular disease, chronic obstructive pulmonary disease, previous MI, cerebrovascular events, and prior cardiac intervention. Hypertension, diabetes, and dyslipidemia were defined according to the stated blood pressure, glucose, glycated haemoglobin (HbA1c), and lipid cutoffs. BMI was calculated as weight in kilograms divided by height in meters squared and classified according to WHO categories. Family history was defined as STEMI in a first-degree relative before 55 years in men or before 65 years in women. Clinical presentation, including chest pain, dyspnea, diaphoresis, nausea, vomiting, heart failure, cardiogenic shock, blood pressure, pulse rate, and symptom-to-presentation time, was documented. Hemoglobin, serum creatinine, white blood cell (WBC) count, platelet count, troponin, creatine kinase-myocardial band (CK-MB), blood glucose, fasting plasma glucose, HbA1c, and lipid profile were performed from baseline venous samples in the hospital laboratory by standard automated methods. The exact analyzer model was not provided in the protocol and should be added before final submission if required by the journal.

ECG site of STEMI was recorded as anterior, inferior, lateral, or posterior involvement. Bedside echocardiography was performed within 24 hours of admission to assess left ventricular ejection fraction, regional wall motion abnormality, and valvular function. Coronary angiography findings were recorded for the culprit artery, including left anterior descending artery (LAD), right coronary artery (RCA), left circumflex artery (LCX), and left main artery, vessel disease extent, and thrombolysis in myocardial infarction (TIMI) flow grade before and after intervention [13]. Treatment was given according to standard STEMI care, including antiplatelet agents, anticoagulants, beta-blockers, angiotensin-converting enzyme (ACE) inhibitors or angiotensin receptor blockers (ARBs), statins, and reperfusion therapy by fibrinolysis or percutaneous coronary intervention (PCI). Primary PCI, rescue PCI, stent type, coronary artery bypass grafting (CABG) referral, timing of reperfusion, preprocedural and postprocedural ejection fraction (EF), and TIMI grade were documented. Outcomes included acute pulmonary oedema, intubation, atrial fibrillation, ventricular arrhythmia, atrioventricular block, cardiac tamponade, post-MI pericarditis, acute kidney injury (AKI) by Kidney Disease: Improving Global Outcomes (KDIGO) criteria, renal replacement therapy, cardiogenic shock, reinfarction, acute stent thrombosis, cardiopulmonary arrest, contrast-induced nephropathy, mechanical complications, death, and hospital stay. Mechanical complications included free wall rupture, ventricular septal defect, papillary muscle rupture, and acute mitral regurgitation. Patients were followed during hospitalization and up to one month for recorded complications.

Statistical analysis was performed using SPSS version 26.0 (IBM Corporation, Armonk, USA). Quantitative variables were assessed for normality and reported as mean ± SD or median with interquartile range, as appropriate. Categorical variables were presented as frequency and percentage. Young and older STEMI groups were compared using the independent samples t-test for normally distributed continuous variables and the Mann-Whitney U test for non-normal variables. The chi-square test or Fisher’s exact test was applied for categorical variables. Odds ratios with 95% confidence intervals were calculated where applicable. Multivariable binary logistic regression was used to identify independent predictors of in-hospital mortality. A p-value ≤0.05 was considered statistically significant.

## Results

Among 300 STEMI patients, the young group had lower median age and slightly higher BMI than the older group. Male patients were more frequent among young patients, 131 (87.3%) versus 116 (77.3%), while females were more frequent in the older group, 34 (22.7%) versus 19 (12.7%) (χ²=5.16, p=0.023). Smoking was higher in young patients, 57.3% versus 35.3% (χ²=14.60, p<0.001). Diabetes, hypertension, dyslipidemia, chronic obstructive pulmonary disease (COPD), cerebrovascular events, and chronic kidney disease were significantly more frequent in older patients. Family history of cardiovascular disease (CVD) was higher in young patients, 24.0% versus 10.7% (p=0.002). Previous MI and prior cardiac intervention were comparable between groups (Table 1).

**Table 1:** Baseline demographic and clinical characteristics of young and older STEMI patients (n=300). Independent samples t-test was applied for normally distributed continuous variables; Mann-Whitney U test for non-normally distributed continuous variables; chi-square test or Fisher's exact test for categorical variables, as appropriate. 95% CI: 95% confidence interval; BMI: body mass index; df: degrees of freedom; MD: mean difference; OR: odds ratio; CVD: cardiovascular disease; MI: myocardial infarction; COPD: chronic obstructive pulmonary disease. A p-value ≤0.05 was considered statistically significant.

Variable	Young (≤40 years), n=150	Older (>40 years), n=150	Statistical test	Test statistic	p-value	Effect estimate	95% CI
Age (years)	32.50 (29.00 to 36.00)	56.00 (51.00 to 62.00)	Mann-Whitney U	0.00	<0.001	Not applicable	Not applicable
BMI (kg/m²)	26.63 ± 4.45	25.58 ± 4.53	Independent t-test (df=298)	2.02	0.044	MD 1.05	0.03 to 2.07
Pack-years among smokers	11.85 (7.58 to 16.58)	19.80 (13.00 to 25.00)	Mann-Whitney U	1184	<0.001	Not applicable	Not applicable
Male gender	131 (87.3%)	116 (77.3%)	Chi-square (df=1)	5.16	0.023	OR 2.02	1.09 to 3.74
Female gender	19 (12.7%)	34 (22.7%)	Chi-square (df=1)	5.16	0.023	OR 0.49	0.27 to 0.91
Smoking, present/past	86 (57.3%)	53 (35.3%)	Chi-square (df=1)	14.60	<0.001	OR 2.46	1.54 to 3.92
Diabetes mellitus	19 (12.7%)	42 (28.0%)	Chi-square (df=1)	10.89	<0.001	OR 0.37	0.20 to 0.68
Hypertension	21 (14.0%)	47 (31.3%)	Chi-square (df=1)	12.85	<0.001	OR 0.36	0.20 to 0.63
Dyslipidemia	34 (22.7%)	61 (40.7%)	Chi-square (df=1)	11.23	<0.001	OR 0.43	0.26 to 0.71
Family history of CVD	36 (24.0%)	16 (10.7%)	Chi-square (df=1)	9.31	0.002	OR 2.64	1.40 to 5.01
Previous MI	4 (2.7%)	11 (7.3%)	Fisher's exact	Not applicable	0.109	OR 0.35	0.11 to 1.11
COPD	5 (3.3%)	20 (13.3%)	Chi-square (df=1)	9.82	0.002	OR 0.22	0.08 to 0.61
Cerebrovascular events	1 (0.7%)	15 (10.0%)	Fisher's exact	Not applicable	<0.001	OR 0.06	0.01 to 0.46
Prior cardiac intervention	2 (1.3%)	4 (2.7%)	Fisher's exact	Not applicable	0.684	OR 0.49	0.09 to 2.73
Chronic kidney disease	1 (0.7%)	16 (10.7%)	Fisher's exact	Not applicable	<0.001	OR 0.06	0.01 to 0.43

Clinical presentation differed between young and older STEMI patients. Chest pain was more frequent in young patients, 142 (94.7%) versus 132 (88.0%) (χ²=4.21, p=0.040), whereas dyspnea was more common in older patients, 73 (48.7%) versus 34 (22.7%) (χ²=22.10, p<0.0001). Median symptom-to-door time was shorter in young patients, 3.30 hours versus 4.45 hours (U=8827.50, p=0.001), and systolic blood pressure at presentation was lower in the young group, 131.00 versus 142.50 mmHg (U=8041.50, p<0.0001). Diaphoresis, nausea, vomiting, and diastolic blood pressure were comparable between groups (Table 2).

**Table 2:** Clinical presentation characteristics in young versus older STEMI patients (n=300). Independent samples t-test was applied for normally distributed continuous variables; Mann-Whitney U test for non-normally distributed continuous variables; chi-square test for categorical variables. 95% CI: 95% confidence interval; df: degrees of freedom; OR: odds ratio; MD: mean difference; SBP: systolic blood pressure; DBP: diastolic blood pressure. A p-value ≤0.05 was considered statistically significant.

Variable	Young (≤40 years), n=150	Older (>40 years), n=150	Statistical test	Test statistic	p-value	Effect estimate	95% CI
Chest pain	142 (94.7%)	132 (88.0%)	Chi-square (df=1)	4.21	0.040	OR 2.42	1.02 to 5.75
Dyspnea	34 (22.7%)	73 (48.7%)	Chi-square (df=1)	22.10	<0.001	OR 0.31	0.19 to 0.51
Diaphoresis	85 (56.7%)	77 (51.3%)	Chi-square (df=1)	0.86	0.354	OR 1.24	0.79 to 1.95
Nausea	46 (30.7%)	51 (34.0%)	Chi-square (df=1)	0.38	0.537	OR 0.86	0.53 to 1.39
Vomiting	33 (22.0%)	40 (26.7%)	Chi-square (df=1)	0.89	0.346	OR 0.78	0.46 to 1.32
Symptom-to-door time (hours)	3.30 (2.30 to 5.47)	4.45 (2.62 to 8.80)	Mann-Whitney U	8828	0.001	Not applicable	Not applicable
SBP at presentation (mmHg)	131.00 (118.25 to 143.00)	142.50 (127.00 to 155.75)	Mann-Whitney U	8042	<0.001	Not applicable	Not applicable
DBP at presentation (mmHg)	83.06 ± 10.03	84.87 ± 10.29	Independent t-test (df=298)	−1.54	0.125	MD −1.81	−4.11 to 0.50

Older patients showed a less favorable laboratory profile at presentation. Hemoglobin was higher in young patients, 14.36 ± 1.64 versus 12.94 ± 2.01 g/dL (t=6.67, p<0.0001), while serum creatinine was higher in older patients, 1.21 versus 0.88 mg/dL (U=5306.50, p<0.0001). Troponin, CK-MB, random blood glucose, fasting plasma glucose, and HbA1c were also significantly higher in older patients. Total cholesterol, low-density lipoprotein cholesterol (LDL-C), and triglycerides were higher in the older group, whereas high-density lipoprotein cholesterol (HDL-C) was higher in young patients. WBC count did not differ significantly between groups, while platelet count was higher in young patients (Table 3).

**Table 3:** Baseline laboratory parameters in young versus older STEMI patients (n=300). 95% CI: 95% confidence interval; df: degrees of freedom; MD: mean difference; WBC: white blood cell; CK-MB: creatine kinase-myocardial band; FPG: fasting plasma glucose; HbA1c: glycated haemoglobin; LDL-C: low-density lipoprotein cholesterol; HDL-C: high-density lipoprotein cholesterol. A p-value ≤0.05 was considered statistically significant.

Variable	Young (≤40 years), n=150	Older (>40 years), n=150	Statistical test	Test statistic	p-value	Effect estimate	95% CI
Hemoglobin (g/dL)	14.36 ± 1.64	12.94 ± 2.01	Independent t-test (df=298)	6.67	<0.001	MD 1.41	1.00 to 1.83
Serum creatinine (mg/dL)	0.88 (0.75 to 1.06)	1.21 (0.97 to 1.68)	Mann-Whitney U	5306	<0.001	Not applicable	Not applicable
WBC count (×10³/mm³)	11747.84 ± 3662.08	12549.82 ± 3777.20	Independent t-test (df=298)	−1.87	0.063	MD −801.98	−1647.33 to 43.37
Platelet count (×10³/mm³)	260548.22 ± 63726.32	243720.51 ± 68384.57	Independent t-test (df=298)	2.20	0.028	MD 16827.71	1807.94 to 31847.48
Troponin (ng/mL)	3.84 (1.91 to 7.80)	5.76 (2.42 to 11.48)	Mann-Whitney U	9188	0.006	Not applicable	Not applicable
CK-MB (ng/mL)	99.00 (55.23 to 181.35)	148.80 (71.78 to 306.52)	Mann-Whitney U	9107	0.004	Not applicable	Not applicable
Blood glucose (mg/dL)	167.00 (130.90 to 197.20)	187.95 (141.72 to 227.57)	Mann-Whitney U	9056	0.004	Not applicable	Not applicable
FPG (mg/dL)	89.80 (81.83 to 99.80)	102.70 (87.28 to 126.67)	Mann-Whitney U	7836	<0.001	Not applicable	Not applicable
HbA1c (%)	5.40 (5.10 to 5.80)	5.80 (5.50 to 7.40)	Mann-Whitney U	6562	<0.001	Not applicable	Not applicable
Total cholesterol (mg/dL)	198.40 ± 35.61	212.64 ± 37.39	Independent t-test (df=298)	−3.38	0.001	MD −14.24	−22.54 to −5.95
LDL-C (mg/dL)	117.55 (99.45 to 136.47)	135.55 (119.10 to 157.95)	Mann-Whitney U	7500	<0.001	Not applicable	Not applicable
HDL-C (mg/dL)	38.88 ± 7.63	36.98 ± 7.56	Independent t-test (df=298)	2.17	0.031	MD 1.90	0.17 to 3.63
Triglycerides (mg/dL)	141.00 (116.03 to 177.82)	176.70 (135.62 to 224.45)	Mann-Whitney U	7714	<0.001	Not applicable	Not applicable

Among 300 STEMI patients, with 150 patients in each age group, anterior STEMI was more frequent in young patients, 85 (56.7%) versus 57 (38.0%) (χ²=10.48, p=0.001), whereas inferior STEMI was more frequent in older patients, 62 (41.3%) versus 42 (28.0%) (p=0.015). Admission and post-procedural left ventricular ejection fraction (LVEF) were significantly higher in the young group. RWMA was more common in older patients, 144 (96.0%) versus 128 (85.3%) (p=0.002). LAD culprit artery and single-vessel disease were more frequent in young patients, while RCA culprit artery and triple-vessel disease were higher in older patients. LCX, left main disease, double-vessel disease, and TIMI grades were comparable (Table 4).

**Table 4:** ECG, echocardiographic, angiographic and procedural characteristics in young versus older STEMI patients. Chi-square test was applied when expected cell frequency was ≥5; Fisher's exact test when expected cell frequency was <5; Mann-Whitney U test for non-parametric continuous variables. 95% CI: 95% confidence interval; ECG: electrocardiogram; STEMI: ST-segment elevation myocardial infarction; df: degrees of freedom; OR: odds ratio; RWMA: regional wall motion abnormality; LVEF: left ventricular ejection fraction; LAD: left anterior descending artery; RCA: right coronary artery; LCX: left circumflex artery; TIMI: thrombolysis in myocardial infarction. Post-procedural TIMI grade was analyzed after excluding patients who did not undergo coronary intervention. A p-value ≤0.05 was considered statistically significant.

Variable	Young (≤40 years), n=150	Older (>40 years), n=150	Statistical test	Test statistic	p-value	Effect estimate	95% CI
ECG Infarct Territory and Echocardiographic Parameters							
Anterior STEMI	85 (56.7%)	57 (38.0%)	Chi-square (df=1)	10.483	0.001	OR 2.13	1.34 to 3.38
Inferior STEMI	42 (28.0%)	62 (41.3%)	Chi-square (df=1)	5.887	0.015	OR 0.55	0.34 to 0.89
Lateral STEMI	16 (10.7%)	17 (11.3%)	Chi-square (df=1)	0.034	0.854	OR 0.93	0.45 to 1.93
Posterior STEMI	7 (4.7%)	14 (9.3%)	Chi-square (df=1)	2.509	0.113	OR 0.48	0.19 to 1.21
RWMA on echo	128 (85.3%)	144 (96.0%)	Chi-square (df=1)	10.084	0.002	OR 0.24	0.10 to 0.62
Admission LVEF (%)	46.65 (41.35 to 53.08)	43.00 (35.35 to 48.85)	Mann-Whitney U	13854	0.001	Not applicable	Not applicable
Post-procedural LVEF (%)	51.30 (45.40 to 56.15)	46.90 (40.35 to 51.98)	Mann-Whitney U	14088	<0.001	Not applicable	Not applicable
Coronary Angiographic and Procedural Characteristics							
Culprit artery — LAD	81 (54.0%)	58 (38.7%)	Chi-square (df=1)	7.091	0.008	OR 1.86	1.18 to 2.95
Culprit artery — RCA	41 (27.3%)	62 (41.3%)	Chi-square (df=1)	6.520	0.011	OR 0.53	0.33 to 0.87
Culprit artery — LCX	25 (16.7%)	26 (17.3%)	Chi-square (df=1)	0.024	0.878	OR 0.95	0.52 to 1.74
Culprit artery — Left main	3 (2.0%)	4 (2.7%)	Fisher's exact	Not applicable	1.000	OR 0.74	0.16 to 3.39
Single-vessel disease	107 (71.3%)	79 (52.7%)	Chi-square (df=1)	11.092	0.001	OR 2.24	1.39 to 3.61
Double-vessel disease	34 (22.7%)	45 (30.0%)	Chi-square (df=1)	2.079	0.149	OR 0.68	0.41 to 1.15
Triple-vessel disease	9 (6.0%)	26 (17.3%)	Chi-square (df=1)	9.348	0.002	OR 0.30	0.14 to 0.67
Pre-procedural TIMI grade	1.00 (0.00 to 2.00)	1.00 (0.00 to 2.00)	Mann-Whitney U	11312	0.931	Not applicable	Not applicable
Post-procedural TIMI grade	3.00 (2.00 to 3.00)	3.00 (2.00 to 3.00)	Mann-Whitney U	10972	0.158	Not applicable	Not applicable

Reperfusion strategy was comparable between groups. Primary PCI was performed in 90 (60.0%) young and 87 (58.0%) older patients (p=0.725), while fibrinolytic therapy and rescue PCI were identical in both groups. Stent type did not differ significantly. Aspirin, P2Y12 inhibitor, and statin use remained high and comparable. Anticoagulant use was higher in young patients, 141 (94.0%) versus 130 (86.7%) (χ²=4.62, p=0.032), as was beta-blocker use, 133 (88.7%) versus 120 (80.0%) (χ²=4.26, p=0.039). Door-to-balloon and door-to-needle times were significantly shorter in young patients. Angiotensin-converting enzyme inhibitor (ACE-I)/ARB use was not significantly different (Table 5).

**Table 5:** Reperfusion, procedural and in-hospital medical treatment characteristics in young versus older STEMI patients. Categorical variables are presented as frequency (percentage); normally distributed continuous variables as mean ± standard deviation. Chi-square test or Fisher's exact test was applied for categorical variables; independent samples t-test for normally distributed continuous variables. 95% CI: 95% confidence interval; PCI: percutaneous coronary intervention; df: degrees of freedom; OR: odds ratio; ACE-I: angiotensin-converting enzyme inhibitor; ARB: angiotensin receptor blocker; MD: mean difference. †Door-to-balloon time was analyzed among patients who underwent primary PCI only. ‡Door-to-needle time was analyzed among patients who received fibrinolytic therapy only. A p-value ≤0.05 was considered statistically significant.

Variable	Young (≤40 years), n=150	Older (>40 years), n=150	Statistical test	Test statistic	p-value	Effect estimate	95% CI
Primary PCI	90 (60.0%)	87 (58.0%)	Chi-square (df=1)	0.12	0.725	OR 1.09	0.69 to 1.72
Fibrinolytic therapy	54 (36.0%)	54 (36.0%)	Chi-square (df=1)	0.00	1.000	OR 1.00	0.62 to 1.60
Rescue PCI	14 (9.3%)	14 (9.3%)	Chi-square (df=1)	0.00	1.000	OR 1.00	0.46 to 2.18
Drug-eluting stent	79 (52.7%)	71 (47.3%)	Chi-square (df=1)	0.85	0.356	OR 1.24	0.79 to 1.95
Bare-metal stent	11 (7.3%)	16 (10.7%)	Chi-square (df=1)	1.02	0.313	OR 0.66	0.30 to 1.48
Aspirin	146 (97.3%)	145 (96.7%)	Fisher's exact	Not applicable	1.000	OR 1.26	0.33 to 4.78
P2Y12 inhibitor	142 (94.7%)	138 (92.0%)	Chi-square (df=1)	0.86	0.355	OR 1.54	0.61 to 3.89
Anticoagulant	141 (94.0%)	130 (86.7%)	Chi-square (df=1)	4.62	0.032	OR 2.41	1.06 to 5.48
Beta-blocker	133 (88.7%)	120 (80.0%)	Chi-square (df=1)	4.26	0.039	OR 1.96	1.03 to 3.72
ACE-I / ARB	107 (71.3%)	94 (62.7%)	Chi-square (df=1)	2.55	0.110	OR 1.48	0.91 to 2.41
Statin	145 (96.7%)	147 (98.0%)	Fisher's exact	Not applicable	0.723	OR 0.59	0.14 to 2.52
Door-to-balloon time (min)	72.4 ± 20.4	79.8 ± 26.3	Independent t-test (df=175)	−2.09	0.038	MD −7.38	−14.35 to −0.42
Door-to-needle time (min)	31.6 ± 10.1	37.8 ± 12.2	Independent t-test (df=106)	−2.87	0.005	MD −6.19	−10.46 to −1.91

In-hospital complications were more frequent among older STEMI patients. Cardiogenic shock occurred in 21 (14.0%) older patients versus 7 (4.7%) young patients (χ²=7.72, p=0.006), while heart failure was also higher in the older group, 40 (26.7%) versus 16 (10.7%) (χ²=12.65, p<0.001). Older patients had more acute kidney injury, renal replacement therapy, mechanical complications, acute mitral regurgitation, and in-hospital death, 17 (11.3%) versus 6 (4.0%) (p=0.017). Atrial fibrillation was higher in older patients, whereas pulmonary oedema, intubation, ventricular arrhythmias, AV block, reinfarction, stent thrombosis, and cardiac arrest were not significantly different. Median hospital stay was longer in older patients, 11.00 versus 6.00 days (p<0.0001) (Table 6).

**Table 6:** In-hospital complications and outcomes in young versus older STEMI patients. STEMI: ST-segment elevation myocardial infarction; df: degrees of freedom; MI: myocardial infarction; CPA: cardiopulmonary arrest.

Variable	Young (≤40 years), n=150	Older (>40 years), n=150	Statistical test	Test statistic	p-value	Effect estimate	95% CI
Acute pulmonary oedema	9 (6.0%)	16 (10.7%)	Chi-square (df=1)	2.14	0.144	OR 0.53	0.23 to 1.25
Endotracheal intubation	11 (7.3%)	17 (11.3%)	Chi-square (df=1)	1.42	0.234	OR 0.62	0.28 to 1.37
Cardiogenic shock	7 (4.7%)	21 (14.0%)	Chi-square (df=1)	7.72	0.006	OR 0.30	0.12 to 0.73
Atrial fibrillation	11 (7.3%)	22 (14.7%)	Chi-square (df=1)	4.12	0.042	OR 0.46	0.21 to 0.99
Ventricular arrhythmias (VT/VF)	16 (10.7%)	27 (18.0%)	Chi-square (df=1)	3.28	0.070	OR 0.54	0.28 to 1.06
Atrioventricular block	7 (4.7%)	12 (8.0%)	Chi-square (df=1)	1.40	0.236	OR 0.56	0.22 to 1.47
Cardiac tamponade	2 (1.3%)	1 (0.7%)	Fisher's exact	Not applicable	1.000	OR 2.01	0.18 to 22.45
Post-MI pericarditis	1 (0.7%)	7 (4.7%)	Fisher's exact	Not applicable	0.067	OR 0.14	0.02 to 1.13
Heart failure	16 (10.7%)	40 (26.7%)	Chi-square (df=1)	12.65	<0.001	OR 0.33	0.17 to 0.62
Acute kidney injury	7 (4.7%)	24 (16.0%)	Chi-square (df=1)	10.40	0.001	OR 0.26	0.11 to 0.62
Renal replacement therapy	0 (0.0%)	6 (4.0%)	Fisher's exact	Not applicable	0.030	Not estimable	Not estimable
Re-infarction	8 (5.3%)	13 (8.7%)	Chi-square (df=1)	1.28	0.258	OR 0.59	0.24 to 1.48
Acute stent thrombosis	1 (0.7%)	2 (1.3%)	Fisher's exact	Not applicable	1.000	OR 0.50	0.04 to 5.54
Cardiac arrest/CPA	11 (7.3%)	18 (12.0%)	Chi-square (df=1)	1.87	0.171	OR 0.58	0.26 to 1.27
Contrast-induced nephropathy	7 (4.7%)	6 (4.0%)	Chi-square (df=1)	0.08	0.777	OR 1.17	0.39 to 3.58
Mechanical complications	5 (3.3%)	19 (12.7%)	Chi-square (df=1)	8.88	0.003	OR 0.24	0.09 to 0.65
Free wall rupture	2 (1.3%)	3 (2.0%)	Fisher's exact	Not applicable	1.000	OR 0.66	0.11 to 4.02
Ventricular septal defect	3 (2.0%)	9 (6.0%)	Fisher's exact	Not applicable	0.138	OR 0.32	0.08 to 1.21
Papillary muscle rupture	2 (1.3%)	4 (2.7%)	Fisher's exact	Not applicable	0.684	OR 0.49	0.09 to 2.73
Acute mitral regurgitation	0 (0.0%)	7 (4.7%)	Fisher's exact	Not applicable	0.015	Not estimable	Not estimable
In-hospital death	6 (4.0%)	17 (11.3%)	Chi-square (df=1)	5.70	0.017	OR 0.33	0.12 to 0.85
Hospital length of stay (days)	6.00 (4.00 to 8.00)	11.00 (8.00 to 13.75)	Mann-Whitney U	3882	<0.001	Not applicable	Not applicable

Multivariable logistic regression was performed using a parsimonious five-variable model because 23 in-hospital deaths occurred among 300 patients, giving 4.6 events per variable. The model was statistically significant, with likelihood ratio χ² (5) =53.97 and p<0.0001. Model performance was acceptable, with McFadden pseudo-R² of 0.332, Nagelkerke R² of 0.394, AUC of 0.895, AIC of 120.36, BIC of 142.58, and satisfactory calibration on Hosmer-Lemeshow testing, χ² (8) =5.35, p=0.720. Mechanical complications were the strongest independent predictor of in-hospital death, followed by ventricular arrhythmias, cardiogenic shock, and acute kidney injury. The older age group was not independently associated with mortality after adjustment for these clinical complications. These findings should be interpreted cautiously, given the exploratory nature of the analysis (Table 7).

**Table 7:** Multivariable logistic regression for independent predictors of in-hospital mortality in young versus older STEMI patients. The model included five predictor variables selected on clinical grounds, with 23 in-hospital deaths yielding an events-per-variable ratio of 4.6. AOR: adjusted odds ratio; 95% CI: 95% confidence interval; VT: ventricular tachycardia; VF: ventricular fibrillation; B: regression coefficient; SE: standard error. A p-value ≤0.05 was considered statistically significant.

Predictor variable	Regression coefficient (B)	Standard error	Wald χ² (df=1)	p-value	Adjusted OR	95% CI
Mechanical complications	2.575	0.641	16.16	<0.001	13.13	3.74 to 46.08
Ventricular arrhythmias (VT/VF)	2.358	0.598	15.55	<0.001	10.57	3.27 to 34.11
Cardiogenic shock	1.833	0.710	6.66	0.010	6.25	1.55 to 25.13
Acute kidney injury	1.577	0.719	4.81	0.028	4.84	1.18 to 19.80
Older age group (>40 years)	0.144	0.574	0.06	0.801	1.16	0.38 to 3.56

## Discussion

The principal finding of this study was that young and older STEMI patients represented two clinically different risk patterns. Young patients were more frequently male, had higher smoking exposure, and had more family history of cardiovascular disease, while older patients had a higher burden of diabetes mellitus, hypertension, dyslipidemia, chronic kidney disease, and previous vascular illness. The anatomical pattern also differed, with young patients showing more anterior STEMI, LAD involvement, and single-vessel disease, whereas older patients showed more RCA involvement, triple-vessel disease, lower LVEF, and more RWMA. In-hospital outcomes were less favorable in older patients, particularly heart failure, AKI, cardiogenic shock, mechanical complications, longer admission, and mortality. After adjustment, mortality was mainly related to acute complications rather than age group alone.

The demographic and baseline profile in this study was largely aligned with previous age-based STEMI comparisons. Young patients showed marked male predominance, 87.3%, with females representing only 12.7%, a pattern close to reports where male sex accounted for most young STEMI presentations [6,14,15]. Smoking was also substantially more frequent in young patients, 57.3% versus 35.3%, which supports previous observations that tobacco exposure remains the dominant modifiable risk factor in early STEMI [8,16]. The higher smoker-only pack-year exposure among older smokers should not be viewed as contradictory, because older age allows longer cumulative exposure despite lower smoking prevalence. Family history was more frequent in young patients, 24.0% versus 10.7%, supporting the contribution of premature familial risk when metabolic disease burden is lower [14,17]. In contrast, diabetes, hypertension, dyslipidemia, and chronic kidney disease (CKD) were more common in older patients, consistent with progressive cardiometabolic exposure over time [12,18,19]. The higher dyslipidemia rate in older patients differed from studies reporting more newly diagnosed dyslipidemia among young patients, probably because documented history and newly measured lipid abnormality are not equivalent [10,20,21].

Clinical presentation differed between age groups, with young patients more often presenting with chest pain and older patients more often presenting with dyspnea. The shorter symptom-to-door interval in young patients, 3.30 versus 4.45 hours, was comparable to earlier reports showing shorter total ischemic time in younger STEMI patients [21,22]. This difference is likely related to more typical ischemic pain in young patients, which may prompt earlier hospital presentation. In contrast, dyspnea was markedly higher in older patients, 48.7% versus 22.7%, consistent with studies showing more atypical symptoms, digestive complaints, and delayed presentation in older adults [12,23-25].

Laboratory findings further supported the higher-risk profile of older STEMI patients. Older patients had lower hemoglobin and higher serum creatinine, indicating reduced oxygen-carrying capacity and renal reserve, both of which may increase vulnerability to heart failure, contrast-related renal injury, and adverse in-hospital course [14,26]. Higher random glucose, fasting glucose, and HbA1c in older patients were consistent with their greater diabetes burden and with evidence linking hyperglycemia to mortality, reinfarction, heart failure, and cardiogenic shock after STEMI [12,27,28]. Higher troponin and CK-MB in older patients may reflect delayed presentation and more extensive coronary disease. WBC count was not significantly different, possibly because leukocytosis depends on infarct size and sampling time rather than age alone [14,18,29]. ECG, echocardiographic, and angiographic findings supported different disease patterns between age groups. Young patients more often had anterior STEMI, 56.7% versus 38.0%, LAD culprit artery, 54.0% versus 38.7%, and single-vessel disease, suggesting a more focal thrombotic presentation [9,14,22]. This pattern is consistent with evidence that smoking and thrombus-heavy lesions are more frequent in young STEMI, with smoking reported as an independent predictor of LAD involvement in young patients [10]. Older patients had more inferior STEMI, RCA involvement, and triple-vessel disease, reflecting more diffuse coronary atherosclerosis related to diabetes, hypertension, and longer vascular exposure [12,23]. LVEF was better preserved in young patients, while higher RWMA in older patients may reflect previous silent ischemia and delayed presentation [9,16,25].

Reperfusion and procedural management were largely balanced between age groups, with comparable rates of primary PCI, fibrinolysis, rescue PCI, and stent use. Primary PCI was performed in 60.0% of young and 58.0% of older patients, differing from reports where younger patients received invasive management more often, likely because treatment access in the present setting was less age-dependent. Door-to-balloon and door-to-needle times were shorter in young patients, consistent with studies showing shorter ischemic and treatment delays among younger STEMI patients [21,22]. Higher anticoagulant and beta-blocker use in young patients may reflect fewer contraindications, while lower use in older patients may relate to shock, renal dysfunction, bradycardia, or pulmonary disease [12,17,30].

In-hospital outcomes were less favorable in older STEMI patients, with higher rates of heart failure, cardiogenic shock, AKI, mechanical complications, renal replacement therapy, acute mitral regurgitation, longer admission, and death. The differences in heart failure, 26.7% versus 10.7%, cardiogenic shock, 14.0% versus 4.7%, AKI, 16.0% versus 4.7%, and mechanical complications, 12.7% versus 3.3%, were consistent with previous age-based STEMI comparisons reporting greater pump failure, renal injury, and structural complications in older patients [10,12,14,19]. This pattern is explained by higher diabetes, hypertension, renal dysfunction, multivessel disease, and lower LVEF, which reduce tolerance to acute infarction. The higher AKI burden also agrees with evidence linking renal injury after STEMI with mortality and prolonged admission [9,10,16].

In multivariable analysis, mechanical complications showed the strongest association with in-hospital mortality, followed by ventricular arrhythmias, cardiogenic shock, and AKI. The absence of an independent effect of older age after adjustment indicates that mortality was mainly explained by acute clinical complications rather than age category alone. This differs from larger registry analyses in which age remained significant, likely because those models used broader age strata and different adjustment variables [9,16,30]. Ventricular arrhythmias and shock also represent established early mortality pathways [11].

The observed differences support distinct clinical pathways across age groups. Young patients mainly showed smoking-related and familial-risk STEMI, with more focal coronary involvement and better preserved LVEF, while older patients had accumulated diabetes, hypertension, renal dysfunction, and diffuse coronary disease, resulting in lower reserve and greater complication burden. This pattern is biologically plausible because chronic metabolic and renal disease reduces tolerance to acute infarction and increases the risk of pulmonary oedema, shock, and AKI [9,16]. Clinically, young patients require strict secondary prevention and risk-factor screening, whereas older patients require early recognition, rapid reperfusion, careful hydration when appropriate, avoidance of unnecessary nephrotoxic drugs, limited contrast exposure during intervention, and close monitoring for shock, arrhythmias, and AKI [12,23,28].

The study was strengthened by the simultaneous assessment of demographic, clinical, laboratory, ECG, echocardiographic, angiographic, treatment, and in-hospital outcome variables in clearly defined young and older STEMI groups. Use of patient-level data allowed estimation of effect sizes and construction of a parsimonious mortality model, although only 23 deaths occurred. The single-centre design limits generalizability, and the adjusted estimates should be interpreted cautiously because the events-per-variable ratio was below the conventional threshold. Differences in age cutoffs across previous studies also limit direct comparison. Long-term mortality, recurrent infarction, heart failure admission, frailty, bleeding severity, cardiac magnetic resonance (CMR)-defined infarct size, socioeconomic factors and medication adherence were not assessed. These factors should be evaluated in future multicentre studies with longer follow-up.

## Conclusions

Older patients with acute STEMI had a higher comorbidity burden and developed more in-hospital complications than young patients; however, after adjustment, in-hospital mortality was mainly associated with mechanical complications, ventricular arrhythmias, cardiogenic shock, and acute kidney injury rather than age group alone. These findings suggest that early recognition and close monitoring of acute complications may be more important than age alone for identifying high-risk STEMI patients during hospitalization.
